# Risk for Invasive Cancers in Women With Breast Cancer *In Situ*: Results From a Population Not Covered by Organized Mammographic Screening

**DOI:** 10.3389/fonc.2021.606747

**Published:** 2021-03-18

**Authors:** Nena Karavasiloglou, Katarina L. Matthes, Giulia Pestoni, Manuela Limam, Dimitri Korol, Miriam Wanner, Sabine Rohrmann

**Affiliations:** ^1^ Division of Chronic Disease Epidemiology; Epidemiology, Biostatistics and Prevention Institute (EBPI), University of Zurich, Zurich, Switzerland; ^2^ Cancer Registry of the Cantons of Zurich, Zug, Schaffhausen, and Schwyz, University Hospital Zurich, Zurich, Switzerland

**Keywords:** breast cancer *in situ*, invasive cancer, risk, standardized incidence ratio, patients

## Abstract

**Background:**

Even though breast cancer *in situ* (BCIS) incidence has been increasing, the prognosis of BCIS patients has not been extensively investigated. According to the literature, women with BCIS have a higher risk of developing subsequent invasive breast cancer; conflicting information has been reported regarding their potential risk for a subsequent invasive non-breast cancer.

**Methods:**

Data from 1,082 women, whose first-ever cancer diagnosis was primary BCIS between 2003 and 2015 and were living in the canton of Zurich, were used. Standardized incidence ratios (SIRs) were calculated to compare the risk of an invasive breast or non-breast cancer among women with a primary BCIS with the corresponding risk of the adult female population. SIRs were calculated overall and by patient and tumor characteristics. To investigate potential risk factors (e.g., age at diagnosis, treatment) for a subsequent invasive breast or non-breast cancer we used Cox proportional hazards regression models.

**Results:**

BCIS patients had 6.85 times [95% confidence interval (CI): 5.52–8.41] higher risk of being diagnosed with invasive breast cancer compared to the general population. They additionally faced 1.57 times (95% CI: 1.12–2.12) higher risk of an invasive non-breast cancer. The SIRs were higher for women < 50-years old for both invasive breast and non-breast cancer at BCIS diagnosis. Age ≥ 70-years old at BCIS diagnosis was statistically significantly associated with a subsequent invasive non-breast cancer diagnosis.

**Conclusions:**

BCIS patients had a higher risk of being diagnosed with invasive breast and non-breast cancer compared to the general population. Age 70 years or older at BCIS diagnosis was the only risk factor statistically significantly associated with a subsequent invasive non-breast cancer. Our results support the increased risk for subsequent cancers in BCIS patients reported in the literature. Future studies should establish the risk factors for subsequent cancers, highlight the need for intensive monitoring in this population, and help distinguish BCIS patients who could benefit from systemic therapy to prevent distant cancers.

## Introduction

Breast cancer *in situ* (BCIS) is an intraepithelial lesion with malignant potential. Generally, BCIS is considered a non-obligatory precursor or a potential risk factor for invasive breast cancer, depending on the morphological subtype. Women with BCIS rarely report symptoms, and the majority of *in situ* tumors are detected through organized or opportunistic mammographic screening attendance (1).

Even though BCIS incidence has been increasing in the past decades (2–5) and it is estimated that BCIS cases account for up to 20% of the screen-diagnosed breast cancer cases (6), the clinical importance of BCIS remains unclear. Additionally, the prognosis of women diagnosed with BCIS has not been thoroughly investigated (6). Based on the existing literature, women with BCIS have a higher risk of developing a subsequent invasive breast cancer (7–11), but the magnitude of the risk varies considerably (3.4 to 7.2 times higher than the risk in the comparison population). Most of the aforementioned studies have been conducted in regions with established organized mammographic screening programs or where organized mammographic screening programs were established during the study period (9, 12). Investigating the risk for subsequent breast cancer in women with BCIS in regions where only opportunistic screening exists will improve our understanding of the progression of the disease since fewer women are using mammograms in these regions (13, 14). In the absence of organized screening, a smaller, selective number of women will choose to undergo mammography for various reasons (e.g., health-consciousness, family history of breast cancer), meaning that some breast cancer *in situ* cases might not be diagnosed. Reports from Switzerland indicate that a lower proportion of women undergoes mammography in the German-speaking regions, where mainly opportunistic screening exists, compared to the French-speaking regions, where organized screening programs have been established (34.9 *vs*. 77.8% of 50–69 year-olds reported a mammogram in the past 2 years, respectively) (13, 14).

Furthermore, the findings regarding the risk of BCIS patients for subsequent invasive cancer in sites other than the breast are contradicting. A previous Swiss study including data from the Canton of Vaud did not report an increased risk for invasive cancer in sites other than the breast (7) compared to the comparison population, while a study conducted in the Netherlands suggested an increased risk in BCIS patients (10).

We aimed to investigate the risk for a subsequent invasive cancer (breast and non-breast) in BCIS patients in the Canton of Zurich, where no organized mammographic screening program exists. To the best of our knowledge, this is the first study to report on the risk for subsequent invasive cancer after BCIS diagnosis in the German-speaking region of Switzerland.

## Methods

### Population

We used data from women whose first-ever cancer diagnosis was primary BCIS [D05.0–D05.9; International Statistical Classification of Diseases and Related Health Problems, 10th revision (ICD-10)] and occurred between 2003 and 2015 (hereafter referred to as primary BCIS). The data were obtained from the Cancer Registry of the Cantons of Zurich, Zug, Schaffhausen, and Schwyz. A recent publication demonstrated that the data quality from the Cancer Registry was acceptable based on four widely used data quality indicators (comparability, validity, timeliness, and completeness) (15). The Cancer Registry started recording cases for the Cantons of Zug, Schaffhausen, and Schwyz later (2011, 2020, and 2020, respectively), so the present analyses only focus on data from the Canton of Zurich. To be included in the study, patients had to live in the canton of Zurich at the time of diagnosis, even if they were treated in another canton. BCIS with morphological codes [according to the International Classification of Diseases for Oncology, 3rd Edition (ICD-O-3)] 8201/2, 8230/2, 8500/2, 8501/2, 8503/2, 8504/2, and 8507/2 were categorized as ductal carcinoma *in situ*. Cases with ICD-O-3 morphological code 8520/2 were categorized as lobular carcinoma *in situ*, and cancers with ICD-O-3 morphological code 8522/2 as unspecified cancers *in situ*. We excluded from our analyses patients diagnosed with a recorded cancer before the BCIS, patients diagnosed with Paget disease of the breast, and patients for whom invasive breast cancer was diagnosed simultaneously with the BCIS. Patients with unknown laterality of BCIS and those diagnosed with bilateral BCIS were also excluded from our analyses.

Our outcomes of interest were invasive breast cancer diagnosis (C50, ICD-10) and invasive non-breast cancer diagnosis (i.e., all sites except breast and non-melanoma skin cancer combined; C00-C96 and D45–D47, excluding C50, ICD-10). The latter tumors (D45–D47, ICD-10) were included in our analyses as invasive non-breast tumors due to their malignant behavior according to ICD-O-3. Thus, patients diagnosed with a second primary *in situ* cancer were excluded from our analyses (n=39). When looking at the risk of invasive breast cancer, patients treated with double mastectomy (n=3) for their primary BCIS were excluded from the analyses given their very low risk for subsequent invasive breast cancer. However, these patients were retained when looking at the subsequent risk of invasive non-breast cancer.

Time at risk for an invasive cancer was assumed to begin 3 months after primary BCIS and lasted until the date of an invasive cancer diagnosis, date of emigration, date of death or end of follow-up (31st December 2016), whichever came first. Thus, patients with less than 3 months of follow-up after their primary BCIS diagnosis were excluded from our analysis. We obtained patients’ vital status from the Citizen Services Departments of the Canton of Zurich. Our final study population included 1,082 patients with primary BCIS diagnosis (1,079 patients when investigating invasive breast cancer risk).

Given the different treatment schemes for BCIS patients, we only focused on the first available treatment after BCIS diagnosis. Treatment options were grouped into four categories as follows: breast-conserving surgery (including quadrantectomy and tumorectomy, with or without lymph node dissection), mastectomy or surgery not otherwise specified (NOS), radiotherapy, hormonal therapy, or other therapies, and unknown.

### Statistical Analyses

Standardized incidence ratios (SIRs) were calculated to compare the risk of invasive breast or non-breast cancer among women with primary BCIS with the corresponding risk of primary invasive breast or primary invasive non-breast tumor in the adult female population of the canton of Zurich. The observed number of invasive breast or non-breast cancers was counted among the index cohort by year and age group at diagnosis (<50, 50–59, 60–69, ≥ 70 years old). The expected number of invasive cancers was estimated among the comparison cohort by multiplying the person-time of follow-up with the corresponding age- and period-specific incidence rates. Ninety-five percent of confidence intervals (CIs) for the SIRs were estimated by assuming Poisson distribution for the observed cases and using Wald's normal-approximation [*popEpi* package in R (16)]. Stratified analyses by morphological subtype, laterality and treatment of primary BCIS, year of primary BCIS diagnosis, age group at primary BCIS diagnosis, and calendar period of primary BCIS diagnosis were performed. Given the small number of patients with unspecified BCIS (n=7), the separate results for this morphological subtype are not shown.

We used univariate Cox proportional hazards regression models to investigate which factors are associated with the risk of being subsequently diagnosed with invasive breast or non-breast cancer [*survival* package in R (17)]. Entry time started 3 months after a participant’s BCIS diagnosis and exit time was defined as the date of diagnosis of subsequent cancer. Participants who were not diagnosed with a subsequent cancer were censored on the date of loss to follow-up, end of follow-up (31st December 2016), or death, whichever came first. Age at BCIS diagnosis, treatment, morphological subtype and laterality of the initial BCIS tumor, as well as sociodemographic factors (nationality and marital status) were investigated as potential risk factors for subsequent invasive cancer diagnosis. Analyses were conducted separately for the risk of invasive breast and non-breast cancer. Results were presented as hazard ratios (HRs) and 95% CIs.

We conducted sensitivity analyses, retaining only women who had at least 6 months of follow-up. All analyses were performed in R (version 3.5.0, R Foundation for Statistical Computing, Vienna, Austria) and significance levels were set at alpha = 0.05.

## Results

Description of the study population and baseline characteristics are shown in **Table 1**. The most frequent invasive cancer diagnoses in BCIS patients were invasive breast, gynecological, or colorectal cancers. Of BCIS patients, those subsequently diagnosed with invasive non-breast cancer (i.e., all sites except breast and non-melanoma skin cancer combined) were older at the time of their initial BCIS diagnosis than both patients who were not subsequently diagnosed with invasive cancer and those subsequently diagnosed with invasive breast cancer.

**Table 1:** Characteristics of study participants based on whether or not they received a second primary cancer diagnosis during the study period.^1^

	No second primary diagnosis (n=950)	Second primary diagnosis (n=132)		
		Invasive breast cancer (n=91)	Invasive non-breast cancer (n=41)
Age at diagnosis of primary BCIS (years, mean ± SD)	57.2 ± 11.2	56.8 ± 11.8	63.5 ± 13.1
Time period of primary BCIS diagnosis, n (%)			
2003–2009	433 (45.6)	59 (64.8)	29 (70.7)
2010–2015	517 (54.4)	32 (35.2)	12 (29.3)
Treatment of primary BCIS, n (%)			
Breast-conserving surgery	639 (67.3)	54 (59.3)	22 (53.7)
Mastectomy or Surgery, NOS^2^	188 (19.8)	17 (18.7)	9 (22.0)
Radiotherapy, Hormonal therapy or Other therapy	82 (8.6)	12 (13.2)	7 (17.1)
Unknown	41 (4.3)	8 (8.8)	3 (7.3)
Morphological subtype of primary BCIS, n (%)			
Ductal	863 (90.8)	80 (87.9)	35 (85.4)
Lobular	81 (8.5)	11 (12.1)	5 (12.2)
Unspecified	6 (0.6)	–	1 (2.4)
Laterality of primary BCIS, n (%)			
Right	440 (46.3)	49 (53.8)	19 (46.3)
Left	510 (53.7)	42 (46.2)	22 (53.7)
Marital status, n (%)			
Never married, widowed, divorced, or Separated	254 (26.7)	22 (24.2)	17 (41.5)
Married or living with partner	451 (47.5)	54 (59.3)	16 (39.0)
Unknown	245 (25.8)	15 (16.5)	8 (19.5)
Nationality, n (%)			
Swiss	684 (72.0)	71 (78.0)	33 (80.5)
Non-Swiss	135 (14.2)	14 (15.4)	3 (7.3)
Unknown	131 (13.8)	6 (6.6)	5 (12.2)

^1^BCIS, breast cancer in situ; NOS, not otherwise specified; SD, standard deviation.

^2^Excluding double mastectomy.

The overall age-standardized incidence rate (ASR) for primary invasive breast cancer in the adult female population of the Canton of Zurich during our study period (2003–2016) was 104.2 per 100,000 person-years. The corresponding ASR for primary invasive non-breast cancer (i.e., all sites except breast and non-melanoma skin cancer combined) was 195.7 per 100,000 person-years.

The results of the risk for subsequent invasive breast cancer after BCIS diagnosis compared to the risk of the general adult female population of Zurich for primary invasive breast cancer are shown in **Table 2**. After 6,362 person-years, BCIS patients had 6.85 (95% CI: 5.52–8.41) times higher risk of receiving an invasive breast cancer diagnosis compared to the general adult female population of Zurich. Women diagnosed with BCIS before the age of 50 had higher SIR (21.74, 95% CI: 14.69–32.18) for invasive breast cancer. Higher SIR was also observed for women diagnosed with BCIS in their right breast (7.96, 95% CI: 6.01–10.53); however, the CIs of the SIRs of women diagnosed with BCIS in their right breast, compared to those diagnosed with BCIS in their left breast were overlapping. The SIRs did not vary considerably by morphological subtype or treatment of the initial BCIS (**Table 2**).

**Table 2:** Standardized incidence ratios (SIRs) for an invasive cancer diagnosis (breast or non-breast) after diagnosis of primary breast cancer *in situ* (BCIS) and its 95% confidence interval (CI), overall and by patient and tumor characteristics.^1^

	Invasive breast cancer		Invasive non-breast cancer	
	O/E	SIR (95% CI)	O/E	SIR (95% CI)
Overall	91/13.28	6.85 (5.52–8.41)	41/26.18	1.57 (1.12–2.12)
Morphological subtype of primary BCIS				
Ductal	80/11.80	6.78 (5.45–8.44)	35/23.44	1.49 (1.07–2.08)
Lobular	11/1.33	8.26 (4.58–14.92)	5/2.49*	2.01 (0.84–4.83)
Treatment of primary BCIS				
Breast-conserving surgery	54/7.96	6.79 (5.20–8.86)	22/15.53	1.42 (0.93–2.15)
Mastectomy or Surgery, NOS	17/2.85	5.96 (3.71–9.59)	9/5.88	1.53 (0.80–2.94)
Radiotherapy, hormonal therapy, or other therapies	12/1.87	6.40 (3.63–11.27)	7/3.58	1.96 (0.93–4.11)
Unknown	8/0.60	13.36 (6.68–26.71)	3/1.19	2.51 (0.81–7.79)
Laterality of primary BCIS				
Right	49/6.16	7.96 (6.01–10.53)	19/12.19	1.56 (0.99–2.44)
Left	42/7.12	5.90 (4.36–7.98)	22/13.99	1.57 (1.04–2.39)
Age at diagnosis of primary BCIS				
< 50 years	25/1.15	21.74 (14.69–32.18)	6/1.49	4.02 (1.81–8.96)
50–59 years	28/4.60	6.09 (4.20–8.82)	9/7.08	1.27 (0.66–2.44)
60–69 years	23/4.48	5.13 (3.41–7.72)	13/8.47	1.53 (0.89–2.64)
≥ 70 years	15/3.05	4.92 (2.97–8.16)	13/9.14	1.42 (0.83–2.45)
Time-period of diagnosis of primary BCIS				
2003–2009	59/9.24	6.39 (4.95–8.24)	29/18.14	1.60 (1.11–2.30)
2010–2015	32/4.04	7.92 (5.60–11.20)	12/8.04	1.49 (0.85–2.63)

^1^BCIS, breast cancer in situ; CI, confidence interval; E, expected number of cases; NOS, not otherwise specified; O, observed number of cases. *1 patient diagnosed with BCIS of unspecified morphological subtype. Given the low count in the Unspecified subtype results are not shown separately for it.

Regarding the risk for invasive non-breast cancer, after 6,371 person-years, BCIS patients had 1.57 (95% CI: 1.12–2.12) times higher risk of being diagnosed with invasive non-breast cancer, compared to the risk of primary invasive non-breast cancer in the general adult female population of Zurich. Women diagnosed with BCIS before the age of 50 had higher SIR (4.02, 95% CI: 1.81–8.96) for invasive non-breast cancer; however, the CI of the SIR were overlapping with those of other age groups. The risk did not vary considerably by morphological subtype, laterality, treatment of the initial BCIS, or time-period of the BCIS diagnosis (**Table 2**).

The association between sociodemographic and tumor characteristics and risk for a subsequent invasive (breast or non-breast) cancer is shown in **Table 3**. In the univariate Cox models, older age at BCIS diagnosis (≥ 70 years old) was statistically significantly associated with increased risk for a subsequent invasive non-breast cancer (HR: 3.34, 95% CI: 1.43–7.82), but not for subsequent invasive breast cancer (HR: 1.21, 95% CI: 0.65–2.27). No associations between morphological subtype, treatment, laterality, time-period of diagnosis (2003–2009 *vs*. 2010–2015), or age at diagnosis of the primary BCIS diagnosis were seen with either invasive breast or non-breast cancer risk. Sensitivity analyses excluding women with less than 6 months of follow-up did not alter our results (data not shown).

**Table 3:** Cox regression (univariate analyses) of subsequent invasive cancer diagnosis by potential risk factors following *in situ* breast cancer diagnosis.^1^

	Invasive breast cancer	Invasive non-breast cancer
	HR (95% CI)	HR (95% CI)
*Tumor characteristics*		
Morphological subtype of primary BCIS		
Ductal	Ref.	Ref.
Lobular	1.14 (0.61–2.15)	1.14 (0.44–2.90)
Treatment of primary BCIS		
Breast-conserving surgery	Ref.	Ref.
Mastectomy or surgery, NOS	0.88 (0.51–1.53)	1.07 (0.49–2.34)
Radiotherapy, hormonal therapy, or other therapies	1.05 (0.56–1.98)	1.31 (0.55–3.10)
Unknown	1.97 (0.94–4.14)	1.74 (0.52–5.80)
Laterality of primary BCIS		
Right	Ref.	Ref.
Left	0.75 (0.49–1.13)	1.02 (0.55–1.88)
Time-period of diagnosis of primary BCIS		
2003–2009	Ref.	Ref.
2010–2015	1.02 (0.64–1.62)	0.99 (0.46–2.11)
*Patient characteristics*		
Age at diagnosis of primary BCIS		
< 50 years	1.21 (0.71–2.08)	0.89 (0.32–2.51)
50–59 years	Ref.	Ref.
60–69 years	1.21 (0.70–2.10)	2.16 (0.92–5.04)
≥ 70 years	1.21 (0.65–2.27)	3.34 (1.43–7.82)
Marital status		
Married or living with partner	Ref.	Ref.
Never married, widowed, divorced, or separated	0.71 (0.43–1.16)	1.66 (0.84–3.28)
Unknown	0.51 (0.29–0.90)	0.86 (0.37–2.00)
Nationality		
Swiss	Ref.	Ref.
Non-Swiss	1.22 (0.69–2.17)	0.60 (0.18–1.95)
Unknown	0.37 (0.16–0.86)	0.63 (0.25–1.62)

^1^BCIS, breast cancer in situ; CI, confidence interval; HR, hazard ratio; NOS, not otherwise specified. Given the low count of patients diagnosed with BCIS of unspecified morphological subtype, results are not shown separately for that subtype.

## Discussion

In our study, we observed an increased risk of a subsequent invasive breast or non-breast cancer diagnosis in BCIS patients compared to the general adult female population of Zurich. Older age at primary BCIS diagnosis was associated with the risk of a subsequent invasive non-breast cancer, but not with subsequent invasive breast cancer.

Overall, our findings suggest that BCIS patients have approximately 6.9 times higher risk for invasive breast cancer compared to the general adult female population of Zurich. These results are in line with previous Swiss and international studies suggesting an increased risk of a subsequent invasive breast cancer in BCIS patients (7–11, 18). In the literature, the risk for subsequent invasive breast cancer in BCIS patients ranges from 3.4 to 7.2 times higher compared to the risk in the comparison population. Our estimates were at the higher end of those reported in the literature, potentially reflecting the lack of organized screening programs in our region (i.e., we are identifying less *in situ* tumors compared to regions with organized screening programs). Some studies looking at the SIRs in regions where organized mammographic screening programs have been implemented suggest that the SIRs are high before and during the implementation of the screening programs, but start decreasing long after implementation (10, 12). It is assumed that with screening in place, all BCIS will be detected, not only those in specific women who undergo elective screening, and treated early, before the detection of invasive components. Based in a previous Swiss study, fewer women reported having had a mammography in the past 2 years in the German-speaking regions compared to the French-speaking regions (34.9 *vs*. 77.8% of 50–69 year olds, respectively) (13).

Another potential explanation for our findings is that the *in situ* lesions in our study population had progressed to higher grade before detection. Our previous findings suggest that one-third of the breast cancer *in situ* lesions in the Canton of Zurich were of high grade (2), indicating that *in situ* tumors in our study population progressed to higher grade before detection. Previous studies suggest that BCIS gradually evolves from low grade, well-differentiated lesions to high grade, poorly differentiated lesions by acquiring genetic mutations (19).

Regarding the risk of invasive non-breast cancer after BCIS diagnosis, the findings in the literature are contradicting. While a Swiss study reported no increased risk for invasive non-breast cancer in BCIS patients (7), a study conducted in the Netherlands suggested a 1.4 times increased risk (10). Our findings are in line with the latter study, adding to the literature suggesting that BCIS patients are at higher risk for both invasive breast and invasive non-breast cancer after their initial BCIS diagnosis. This increased risk could be attributed to increased surveillance in BCIS patients after their cancer diagnosis, compared to the general population, or could be an indication of genetic or lifestyle factors that are associated with increased risk for both BCIS and invasive cancers.

Older age at BCIS diagnosis (≥ 70 years old) was statistically significantly associated with a subsequent invasive non-breast cancer, but not with a subsequent invasive breast cancer diagnosis in the univariate Cox models. The lack of statistically significant associations with the majority of the factors investigated in this study could suggest that basic primary tumor or patient characteristics might not be strongly associated with subsequent invasive cancers after an initial BCIS diagnosis. Some studies in the literature also failed to detect such associations. However, larger studies have reported associations of younger age at diagnosis of the initial BCIS, race, or family history for breast cancer with subsequent cancer risk (9, 12, 20). The direction of our results regarding younger age at diagnosis for invasive breast cancer are consistent with those reported in the literature. Another possible explanation is that the classification we used for some factors (e.g., primary BCIS treatment grouped in four categories) did not allow for possible associations to be detected as statistically significant. This classification was used to accommodate the low number of cases in the Canton of Zurich. Thus, future projects should aim to include larger BCIS populations to be able to detect potential associations between primary tumor characteristics, as well as patient characteristics and subsequent invasive cancer diagnoses.

Our study had several strengths. Given the high registry coverage in the Canton of Zurich, we are confident that we capture almost all incident cancer cases in the canton (15). Additionally, medical and treatment information, as well as patient and tumor characteristics were available for a high proportion of our study population, allowing us to stratify the SIRs for these factors while also assessing them as potential risk factors for subsequent invasive cancer diagnoses in the Cox models.

However, this study also had some limitations. Information on potential risk factors such as family history for breast cancer, genetic polymorphisms, parity, and age at first full-term pregnancy that have been associated with invasive cancer risk in previous studies was not available. Additionally, due to the small number of subsequent invasive breast cancer cases, we could not investigate separately the risk of ipsilateral vs. contralateral invasive breast cancer. As with most studies focusing on outcomes in cancer patients, the intensive monitoring of patients after their initial BCIS diagnosis, may have increased the probability of detecting the invasive cancer (i.e., selective surveillance bias) in them. Women included in our study might have attended opportunistic mammographic screening for different reasons (e.g., health-consciousness, family history for breast cancer), which could differentiate them from the general population. Unfortunately, the lack of information on the reasons for screening attendance and family history for breast cancer did not allow us to explore this further. Finally, since not all *in situ* cancer registration is mandatory (i.e., in sites other than the breast), we cannot exclude the possibility that some women in our study might have previously had an *in situ* cancer of another site that was not registered, and thus, they should have been excluded from our analyses. However, since the most frequent *in situ* cancers are registered by the cancer registry, a very low number of women might be falsely included in our analyses, if any at all.

In summary, BCIS patients in the canton of Zurich faced approximately 6.9 times higher risk of invasive breast cancer compared to the general population. They additionally faced 1.6 times higher risk of invasive cancer in sites other than the breast. Of all the potential risk factors for a subsequent invasive (breast or non-breast) cancer tested, only age equal to or greater than 70 years old at BCIS diagnosis was statistically significantly associated with a subsequent invasive non-breast cancer diagnosis. Our results support the increased risk for subsequent cancers in BCIS patients reported in the literature. Future studies should establish the risk factors for subsequent cancers, highlight the need for intensive monitoring in this population, and help distinguish BCIS patients who could benefit from systemic therapy to prevent distant cancers.

## Data Availability Statement

The datasets generated during and/or analyzed during the current study are not publicly available, but are available from the corresponding author upon reasonable request.

## Ethics Statement

Ethical review and approval were not required for the study on human participants in accordance with the local legislation and institutional requirements. Written informed consent for participation was not required for this study in accordance with the national legislation and the institutional requirements.

## Author Contributions

Conception and design: NK and SR. Data acquisition: ML, DK, and MW. Analyzing the data: NK, KM, and GP. Interpretation of the data: NK and SR. Drafting the manuscript: NK. Critically revising the manuscript: NK, KM, GP, ML, DK, MW, and SR. All authors contributed to the article and approved the submitted version.

## Funding

This work was supported by Krebsforschung Schweiz under Grant KFS-4114-02-2017 to SR. The funder had no role in study design, data collection and analysis, decision to publish, or preparation of the manuscript.

## Conflict of Interest

The authors declare that the research was conducted in the absence of any commercial or financial relationships that could be construed as a potential conflict of interest.
